# Gender Differences in Cancer Susceptibility: An Inadequately Addressed Issue

**DOI:** 10.3389/fgene.2012.00268

**Published:** 2012-11-28

**Authors:** M. Tevfik Dorak, Ebru Karpuzoglu

**Affiliations:** ^1^Robert Stempel College of Public Health and Social Work, Florida International UniversityMiami, FL, USA; ^2^College of Veterinary Medicine, University of GeorgiaAthens, GA, USA

**Keywords:** genetic predisposition to disease, sex factors, genetic epidemiology, sex hormones, sex chromosomes, cancer susceptibility

## Abstract

The gender difference in cancer susceptibility is one of the most consistent findings in cancer epidemiology. Hematologic malignancies are generally more common in males and this can be generalized to most other cancers. Similar gender differences in non-malignant diseases including autoimmunity, are attributed to hormonal or behavioral differences. Even in early childhood, however, where these differences would not apply, there are differences in cancer incidence between males and females. In childhood, few cancers are more common in females, but overall, males have higher susceptibility. In Hodgkin lymphoma, the gender ratio reverses toward adolescence. The pattern that autoimmune disorders are more common in females, but cancer and infections in males suggests that the known differences in immunity may be responsible for this dichotomy. Besides immune surveillance, genome surveillance mechanisms also differ in efficiency between males and females. Other obvious differences include hormonal ones and the number of X chromosomes. Some of the differences may even originate from exposures during prenatal development. This review will summarize well-documented examples of gender effect in cancer susceptibility, discuss methodological issues in exploration of gender differences, and present documented or speculated mechanisms. The gender differential in susceptibility can give important clues for the etiology of cancers and should be examined in all genetic and non-genetic association studies.

Gender differences in susceptibility to a disease is a very useful piece of information that can be used to develop a causal hypothesis for the disease, or to define subgroups at highest risk for preventive action (Zahm and Fraumeni, 1995). The gender differential in cancer incidence rates is comparable to ethnic and racial disparity in magnitude, and yet, most studies fail to look for it. In genetic epidemiologic studies, if examined, it is common that genders do not have equal susceptibility to diseases. Exploration of gender disparity in disease susceptibility has not become common practice even in diseases that show sex differential in their incidence rates. Here, we will document this phenomenon in cancer susceptibility with examples, discuss possible mechanisms, and review basic methodology for its examination.

## Descriptive Epidemiology of Gender Differences in Cancer Susceptibility

Epidemiology is sometimes considered to be an unhealthy science because of frequently documented inconsistencies in findings and replication failures. It is relatively infrequent that a finding will be sustained in follow-up studies. However, gender differential in cancer occurrence is one very consistent finding in descriptive epidemiology. For a very long-time, it has been recognized that males are more prone to develop cancer, and particularly hematologic malignancies (Ashley, 1969; Pearce and Parker, 2001; Cartwright et al., 2002; Cook et al., 2009; Edgren et al., 2012). Prognosis in childhood cancers is also worse in boys than girls (Eden et al., 2000), and second malignancies occur more frequently in boys who have survived cancer (Devarahally et al., 2003). In adult malignancies, again, males have worse overall survival and higher mortality rates (Molife et al., 2001; Cook et al., 2011).

### Gender differences in united states data

The sex differential in primary cancer susceptibility can be quantified by comparison of incidence rates in males and females. The latest analysis of the National Cancer Institute Surveillance Epidemiology and End Results (SEER) database showed that incidence rates per 100,000 people and age-adjusted to the 2000 US standard population for all cancers in the United States for 2004–2008 are 553.0 for males and 416.5 for females yielding an incidence rate ratio (IRR) of 1.33 for all cancers (Siegel et al., 2012). Although these numbers are inclusive of sex-specific cancers such as breast and prostate, their exclusion does not alter the IRR (1.35). Likewise, the lifetime probability of developing cancer is 44.85% for males, and 38.08% for females. The probability is higher for males despite a shorter life expectancy. The same analysis also showed that the cancer mortality rates were higher in males: 223.0 versus 153.2 (ratio = 1.46). Most importantly, the common cancers have the highest male-to-female (M:F) ratio: colorectal cancers: 1.35; lung and bronchus: 1.52; non-Hodgkin lymphoma (NHL): 1.44; (urinary) bladder: 4.0. Another recent analysis of SEER identified Kaposi sarcoma as having the highest M:F IRR (28.7; Cook et al., 2009). Other than breast cancer, which rarely occurs in males, only a few cancers are more common in females. Gall bladder, anus, and thyroid tumors consistently show an M:F ratio less than 1.0 (Cook et al., 2009). In Table 1, we present sex-specific incidence rates in the US and worldwide for selected cancers.

**Table 1:** Selected cancers with the highest and lowest male-to-female ratios*.

Cancer	Male-to-female ratio	
	SEER (USA)	IARC (world)
Larynx	5.17	6.36
Hypopharynx	4.13	5.75
Kaposi sarcoma	28.7	4.87
Lip	7.16	4.25
Urinary bladder	3.92	4.12
Gall bladder and biliary tract	0.57	0.94
Anus	0.81	0.85
Thyroid	0.39	0.33

*SEER data from Cook et al. (2009), Siegel et al. (2012); GLOBCAN (world) data from Edgren et al. (2012). In SEER data, all ages combined, the cancer burden was higher in males than females for total cancer (male-to-female incidence rate ratio = 1.37; 95% CI = 1.37–1.38. For the world data, all sex differences were statistically significant (*P* < 0.0001).

### Gender differences are seen worldwide

The increased M:F ratio for incident cancer is not unique to a particular country, population, or region. The analysis of age- and sex-specific cancer incidence data from Cancer Incidence in Five Continents provided by the International Agency for Research on Cancer (IARC) documented the universal nature of the sex disparity in cancer (Edgren et al., 2012). In 32 of 35 cancer sites, males had a higher incidence rates consistently across geographical regions. The three exceptions were thyroid, gallbladder, and anus cancer that had higher incidence rates in females. The most drastic occurrences of male excess were noted for laryngeal and hypopharyngeal cancer, which showed around sixfold excess in males. In this global dataset, sex differences were statistically significant at all sites (*P* < 0.0001). When age in adult life is taken into account, some variation is evident, especially for skin cancer and salivary gland cancer with both becoming more common in males in increasing age groups. NHL, along with its globally consistent high M:F ratio, showed consistency also in all age groups: M:F ratio equals 1.42 with little variation in age groups (range = 1.36–1.59).

### Childhood cancers show gender differences too

In childhood cancer, males are again at a higher risk than females. The sex differential in the incidence of childhood cancer is well established and consistent worldwide (Ashley, 1969; Greenberg and Shuster, 1985; Linet and Devesa, 1991; Pearce and Parker, 2001; Cartwright et al., 2002; Desandes et al., 2004). The M:F ratio for all incident cancers is around 1.2. Exceptions to the male excess in childhood cancer include infant leukemia, thyroid carcinoma, malignant melanoma, and alveolar soft part sarcoma. As in adults, NHL shows a stable male excess in all age groups during childhood and adolescent period (range = 1.7–3.2), whereas Hodgkin lymphoma (HL) shows an interesting age-dependent variation in its M:F ratio (Ries et al., 1999). The overall incidence of HL in children is greater in females than in males (M:F ratio = 0.8), but the sex distribution is age-dependent, with the striking M:F ratio in HL in younger ages when the disease is rare reversing for adolescents when it becomes more common (Spitz et al., 1986). The SEER data from 1990 to 1995 period show M:F ratios of 5.3 (<5 year), 1.4 (5–9 year), 1.1 (10–14 year), and 0.8 (15–19; Ries et al., 1999).

### Gender differences in infections are similar

There are other conditions that males and females show different levels of susceptibility. Any corollary finding may also help develop etiologic hypotheses. The most relevant one is the increased incidence of infections in males in humans and animals, and in controlled animal experiments (Washburn et al., 1965; Schlegel and Bellanti, 1969; Purtilo and Sullivan, 1979; Green, 1992; Read et al., 1997; Klein, 2000; Wells, 2000). Women also survive sepsis at higher rates than men (Schroder et al., 2000). This commonality between cancer and infections suggest that the difference may lie in the male and female immune system since immune surveillance mechanisms are similar against infectious agents and cancer cells. Although infections are generally more common in males, morbidity and mortality rates may be higher in females for some infections such as HIV infection and influenza, especially in those where immune activation and cytokine storm have deleterious effects on prognosis (Klein et al., 2012). The greater severity of post-infectious immunopathologic events in females is due to their ability to generate higher proinflammatory cytokine and chemokine responses than males. This should not be confused with the lower primary susceptibility of females to infections due to enhanced immune response capability.

## Gender-Specific Associations in Cancer Genetic Epidemiology

Not just cancer, but some other common diseases also show some gender differential. It is important to recognize and use it to gain insight into disease biology. In addition to a systematic exploration of the mechanisms underlying physiological differences between sexes using genetical genomics (Li et al., 2008), genetic epidemiology can be used to probe disease biology (Hindorff et al., 2009). Genetic associations with sex-specificity should be able to shed some light on the pathogenesis of diseases showing such associations. While there has been some encouragement by the government agencies such as the US National Institutes of Health and Institute of Medicine, and several journals (Arnold, 2000; Blaustein, 2012), not much progress has been noted in exploring sex-specificity of genetic associations. Most of the papers that reported sex-specific genetic associations have not stood the test of time (Patsopoulos et al., 2007). In a survey of prominent claims of sex differential in genetic associations with common diseases, most of the claims were found to be spurious or insufficiently supported by the data presented.

### Autosomal chromosomes versus sex chromosomes

There seems to be a general consensus that gene expression changes are the most common intermediate phenotype between genetic variants and modification of disease risk (Nica and Dermitzakis, 2008; Cookson et al., 2009; Murphy et al., 2010). It is only natural that any sex differential in gene expression levels should concern genes encoded in sex chromosomes. Even X chromosome genes show variation in their expression among females (Carrel and Willard, 2005). Most studies have found that autosomal genes make up the majority among those with sex-biased expression (Ostrer, 2001; Rinn et al., 2004; Mank et al., 2010; Arnold and Lusis, 2012). Thus, the potential of any genetic variant to yield a gender-specific association does not depend on its genomic location. A recent re-analysis of GWAS top hits for common disorders have yielded gender-specificity for some of them (Liu et al., 2012), despite that only the variants that were found significantly associated in the original overall analyses were included in the re-analysis. It is hoped that these initial results will encourage researchers to re-analyze complete datasets to uncover sex-specific associations, which may have been totally missed if males and females had associations in opposite directions (see next section).

We have screened the literature for sex-specific genetic associations with cancer susceptibility, and provided a list of selected examples in Table 2.

**Table 2:** **Examples of genetic associations with cancer susceptibility exclusive to males or females**.

Cancer	Locus/SNP	PubMed ID
Various cancers	MDM2 (SNP309; accelerated age of diagnosis in females)	17322917
Childhood acute lymphoblastic leukemia (ALL)	MDM2 (SNP309; accelerated age of diagnosis in females)	19837266
Childhood ALL	HLA-DRB4 (rs2395185; risk association in males only)	10397736 (Kennedy et al., unpublished data)
Childhood ALL	HLA-DRA (rs3135388), HLA-C (rs9264942; risk and protective associations in females only)	21067287
Childhood ALL	HSPA1B (rs1061581; stronger protective association in males)	20012387
Childhood ALL	IFNG (rs2069727; protective association in males only)	21067287
Childhood ALL	IRF4 (rs12203592; risk association in males only)	19897031
Childhood ALL	HFE (rs807212; protective association in males only)	19806355
Childhood ALL	CYP1A1 (rs1799814), NAT1/NAT2 (associations in females “CYP21A2/protective” and males “NAT1–NAT2/risk” only)	10953966
Childhood ALL	ARID5B (rs10994982 and rs10740055; risk association in males only)	20460642
Acute myeloblastic and lymphoblastic leukemia	NQO1 (rs1800566) and GSTT1 (deletion; stronger risk association in males)	17339179
Nasopharyngeal cancer	MICA (short tandem repeat polymorphism and deletion; increased MICA*A9 and *A5.1 frequencies in males; increased MICA/HCP5 deletion in males)	16547745; 21536588
Lung cancer	VEGF (haplotypic risk association in males only)	18223238
Lung cancer	MTHFR (risk and protective associations in females only)	15941959
Lung cancer	CYP1A1 (stronger risk association in males)	12594823
Lung cancer	GSTM1 (stronger risk association in females)	9855008
Lung cancer	Chromosome 15q25 region (rs3841324; protective association in females only)	22028403
Upper aerodigestive tract cancers	Chromosome 15q25 region (rs16969968; risk association in females only)	21335511
Colorectal cancer	GSTM1 and GSTM3 (stronger associations in males)	11408349
Colorectal cancer	APOE (risk association in females only)	12529167
Colorectal cancer	NAT2 (risk association in females only)	19107910
Pancreas cancer	NAT1 (risk association in males only)	18499698
Bladder cancer	SULT1A1 (rs9282861; stronger protective association in females)	14643027

## Methodological Issues in Examination of Gender Difference in Cancer Susceptibility

Exploration of gender differences does not require any special method other than those already being used to examine homogeneity of effect size in different strata (effect modification). Treating gender as an environmental factor and testing associations for interaction with gender is one way of identifying associations modified by gender. A significant interaction may indicate either variable strength of the associations, or opposite direction of the associations in males and females. Analysis after stratification by gender clarifies the situation, and unravels any gender-specificity which may otherwise go unnoticed, especially if the association is with decreased risk in one gender, and with increased risk in the other.

### Examination of gender differences is a simple process

In cancer susceptibility, the role played by the environment is much greater than that of genetics (Lichtenstein et al., 2000; Le Marchand, 2005; Hemminki et al., 2006). It is therefore important to remind ourselves that genetic factors are more likely to be modifiers of susceptibility rather than primary determinants of susceptibility (Le Marchand, 2005). It has been suggested that genetic susceptibility studies in cancer would yield more meaningful results if performed in high risk subpopulations (Le Marchand, 2005). Likewise, if either sex has a higher risk as descriptive epidemiology strongly suggests, analyzing males and females separately should produce useful results. Discussions of various issues regarding the subgroup analysis or losing statistical power do not necessarily apply to the analysis of results after stratification by sex. Literature is full of examples unraveling gender-specific associations only after stratifications which were not evident in overall analysis (Table 2). Given that the re-analysis of even the markers already associated with diseases in the overall analysis can show sex-specific associations (Liu et al., 2012), a similar approach for candidate markers even if they do not show any association in the whole sample should be common practice. The benefits of uncovering hidden associations is likely to outweigh any concern over multiple comparisons, which can be handled either statistically by a variety of methods (Rice et al., 2008) or epidemiologically by replication (Hattersley and McCarthy, 2005; Siontis et al., 2010). The concerns over losing statistical power when the sample is split into males and females do not take into account that in a gender-specific association situation, not splitting the sample causes loss of power. With the increasing effect size in the sex which shows the association exclusively, the statistical power will be much higher than that in the pooled sample. Even some of the missing heritability in GWAS (Manolio et al., 2009) may be identified through routine use of sex-specific analysis, as the *post hoc* analysis done in one study suggested (Liu et al., 2012).

### Gender differences can be found frequently from genetic linkage to metabolomics studies

There are published reports on the success of taking gender into account in data analysis. As listed in Table 2, genetic associations may differ between males and females when explored. Likewise, genetic linkage studies have yielded different gender-specific linkage results in inflammatory bowel disease (Fisher et al., 2002) and a number of complex traits such as blood LDL, HDL levels, and systolic blood pressure (Weiss et al., 2006). In the latter study, heritability values also differed for those traits between males and females. It is now becoming a common finding in genetical genomics studies that expression levels of even autosomal genes differ between males and females (van Nas et al., 2009). Most recently, even a metabolomics study revealed gender differences in metabolite levels and their correlations with genetic markers (Mittelstrass et al., 2011). Thus, it is clear that when explored, gender differences are possibly there to be revealed. Gender could and should be considered an environmental variable that can modify both penetrance and expressivity of a wide variety of traits (Weiss et al., 2006).

### Not all gender differences are real

A detailed survey of the biomedical literature for reported gender differences in genetic associations highlighted many prevailing problems (Patsopoulos et al., 2007). Most claims were not documented sufficiently. A common example was finding a smaller *P* value in one group and claiming a gender differential without assessing the difference statistically. This situation may well arise when one gender is underrepresented in the sample and resulting in lower statistical power. Many spurious claims have also been identified. These included claims of sex differences based on comparisons of male and female cases only, or comparisons of a certain genotype ignoring other genotypes of the same locus, or comparisons of one gender against a subgroup of the other. It is therefore important that the search for the gender effect should not be turned into a fishing expedition. It is best just to simply analyze the whole sample for each gender separately, and if there is any indication of a differential, to assess the statistical interaction formally.

### Gender differences appear in different ways

It may sound implausible, but there are examples of genetic associations in opposite directions in each sex (Ober et al., 2008). One of the most remarkable gender-specific associations concerns the recombination rates in the human genome (Kong et al., 2008), which is itself a sexually dimorphic trait (Broman et al., 1998). A GWAS design was used to examine the genetic basis of this recombination rate differences. SNPs in the RNF212 gene showed associations with their statistical significance exceeding genome-wide threshold level both in males and females separately. However, the associations were in opposite directions. The associations observed were real and confirmed in a second study cohort (Kong et al., 2008). The presence of similar robust and replicated associations of IFNG in asthma (Loisel et al., 2011) and RELN in schizophrenia (Shifman et al., 2008) further confirms the reality of gender-specific associations. There are different patterns of gender differences in genetic susceptibility (Ober et al., 2008). The IFNG association in asthma is particularly intriguing in that the association is with the heterozygous genotype, but in opposite directions in males and females (Loisel et al., 2011): heterozygote advantage in females and heterozygote disadvantage in males.

### Statistical adjustment is not sufficient

Although not highlighted by the authors of the survey, a common problem in handling the gender effect on susceptibility is adjusting the results for the gender and declaring no change as evidence for the lack of gender difference in susceptibility. This is commonly done, but it rules out confounding by sex, which is not equivalent to gender difference in susceptibility. If adjustment for gender does not change the results, gender can be ruled out as a confounder, but these results say nothing about possible interaction with gender. To rule out interaction of association results with gender, which is the indication of a gender difference in susceptibility, simple statistical methods are available. These include logistic regression with genotype-gender interaction term (Nick and Campbell, 2007) and tests for homogeneity of odds ratios across different strata (Paul and Donner, 1989). Recently, a permutation-based method to compute gender difference *P* values has been developed and used successfully (Liu et al., 2012).

In summary, to uncover gender differences in genetic association studies, the design should include both males and females in the study sample, the information on gender should be collected or this information should be obtained by genotyping the samples (Gold et al., 2001), and interaction with gender difference should be assessed by stratified analysis. Analysis of males and females separately is the only way to unmask an association that shows gender difference. In a simple case-control study when the outcome is a binary variable, exploration of genotype, and gender interaction by logistic regression is a mostly neglected, but rather straightforward approach. For more complicated designs and for quantitative outcomes, methods developed to analyze gene and environment interactions can be used as if gender was a binary environmental exposure variable (Thomas, 2010).

## Mechanism of Gender Differences in Cancer Susceptibility

In some diseases, the gender disparity can be explained easily: mutations in X-linked immune system genes cause congenital forms of immune deficiencies such as X-linked combined immunodeficiency and Wiskott–Aldrich syndrome almost exclusively in males due to the sex chromosome composition differences between males and females. On the other hand, most smoking-related diseases, like chronic obstructive pulmonary disease, are historically more common in males. It is therefore differences in either an endogenous biological determinant or environmental exposure that cause the disparity. In cancer susceptibility, however, the reasons for the gender difference remain enigmatic (Edgren et al., 2012).

Several factors may contribute to the development of gender disparity in general disease susceptibility, including sex hormones, genetic differences, environmental causes (Zahm and Fraumeni, 1995; Klein, 2000; Cook et al., 2009), gender-linked differences in the skin (Giacomoni et al., 2009), epigenetics (Kaminsky et al., 2006; Gabory et al., 2009), and as more recently suggested, microchimerism (Ober et al., 2008; Ghazeeri et al., 2011), and autophagy (Lista et al., 2011). The common belief is that the male excess in cancer incidence is due to variation in environmental and occupational exposures, including smoking, diet, and sunlight exposure. Environmental exposures indeed dominate overall cancer risk, but environmental variations alone cannot explain the sex differential in cancer risk. Differences in environmental exposures for each cancer that is more common in either sex are known for few cancers but not as a general phenomenon (Zahm and Fraumeni, 1995). Any differential exposure would have to apply to young adults and even children, who have little cumulative exposure to environmental genotoxins. Thus, if not all, some contribution to male excess in cancer is expected to come from genetic factors.

### Physiological differences between males and females

There are known physiological differences that may explain why there is a disparity in the incidence of certain cancers (Table 3). Hepatocellular carcinoma (HCC) is more common in males and one reason for this difference may be higher interleukin-6 (IL-6) levels in males. IL-6 aids progression of HCC and IL-6 production is inhibited by estrogens, which lower the risk of HCC in females (Naugler et al., 2007; Yeh and Chen, 2010). On the other hand, women are less likely than men to develop colon cancer, and estrogen hormone replacement therapy further reduces the incidence of colorectal cancer in postmenopausal women. Collectively, estrogen appears to be protective for colorectal cancer development (Lawrence et al., 2007; Weige et al., 2009). Men are more prone to develop skin cancers including squamous cell carcinomas and melanoma. The skin has gender-specific differences that may explain this disparity (Giacomoni et al., 2009). Ultraviolet exposure and stress induce immunosuppression in the human skin (Streilein et al., 1994), and this effect is stronger in males (Damian et al., 2008; Reeve et al., 2012). The higher incidence of thyroid and gallbladder tumors in women seem to be a natural consequence of higher prevalence of benign thyroid disease and gallbladder stones, which are known to be risk factors for these cancers (Edgren et al., 2012). Why benign thyroid disease and gallbladder stones are more common in females is, however, unknown. The main physiologic differences between males and females are caused by sex hormones, and these will be discussed later.

**Table 3:** **Physiologic and genomic differences between males and females that may be relevant to differential cancer susceptibility**.

Trait	PubMed ID
Transcriptional sexual dimorphism in preimplantation embryos due to lack of X chromosome inactivation at that stage	20501630; 21339284
Greater fetal growth rate and higher birth weight in males	8300805; 17344203
Higher endothelin-1 blood levels in males	8439117
Higher gastrin-releasing peptide receptor (GRPR) levels in females	10620630
Higher leptin levels in females from birth to adulthood	12105281
Higher iron stores in males	21161013
Immunologic functions are stronger in females	517475; 6228595
Immunomodulatory effects of vitamin D(3) are stronger in females than in males	20855882
Gene expression level differences in autosomal as well as sex chromosome genes	10620630; 11641384; 15177028; 15851067; 16469768; 16825664; 17646949; 19190082; 18974276; 20142440; 21858147; 22434084; 19190082; 18156105
Long-range transcriptional regulatory elements associated with chromatin structure (correlating with sex-specific gene expression levels)	20876297
Higher methylation levels in males	17851693
Overall higher recombination rates in female germ cells (except at the telomeres of chromosomes where male recombination rates are higher)	9718341
Differences in alternative splicing	20009012
Higher level of DNA damage in males	12297147
Higher *in vitro* gamma-ray-induced chromatid breaks in males	10856961
Lower DNA repair capacity in females	11058619; 19065660; 18845243; 12917198; 20022891
Higher baseline micronucleus or micronucleated binucleate cell frequency in females	11170240; 9729354
Higher CYP1A1 expression and DNA adduct levels in the lung of female smokers	16557573
Higher lipid peroxidation in females	12142263

The importance of exploring the mechanism of the gender effect in cancer susceptibility is manifold. Besides identifying the subpopulations at higher risk to implement preventive measures, and learning about disease biology for prevention as well as therapeutic interventions, we may also clarify some paradoxical phenomena. Lung cancer provides an interesting example for the last point. In absolute numbers many more men develop and die from lung cancer, but this does not mean they are more susceptible. Studies of molecular mechanisms of lung cancer development in males and females to test the earlier suggestions that females may actually have greater inherent susceptibility to lung cancer, yielded positive results. It has now been widely accepted that although males have higher morbidity and mortality rates for lung cancer, women are at greater risk to develop it (Kiyohara and Ohno, 2010). The higher incidence rate in males is due to higher frequency of smoking.

### Differences in regulation of gene expression between males and females

Since gene expression changes are the most common intermediate phenotype underlying variable disease risk (Nica and Dermitzakis, 2008; Cookson et al., 2009; Murphy et al., 2010), such changes may also be responsible for differential risk between males and females. Sexual dimorphism in gene expression has been repeatedly documented in animals and humans (Shriver et al., 2000; Ostrer, 2001; Rinn et al., 2004; Rinn and Snyder, 2005; Clodfelter et al., 2006; Yang et al., 2006; Isensee and Ruiz Noppinger, 2007; Isensee et al., 2008; Penaloza et al., 2009; van Nas et al., 2009; Mank et al., 2010; Zhang et al., 2011; Arnold and Lusis, 2012; Conforto and Waxman, 2012). This dimorphism extends beyond simple transcript abundance and concerns the organization of gene expression networks (van Nas et al., 2009). In more detailed studies, few autosomal genes are constitutively sex-biased throughout different time points, and a minority of genes change the direction of sex bias (Mank et al., 2010). It may sound implausible, but the most comprehensive sex-specific gene expression studies in mice identified a surprisingly large number of significant differences in gene expression between the sexes, ranging from a few hundred to more than ten thousand in the different tissues examined (Yang et al., 2006). Many of the genes that were identified have been implicated in various common diseases in which disease susceptibility is sex-biased.

Gene expression at the mRNA level does not always correlate with protein expression. What matters is whether sex-specific gene expression differences are translated into the protein level. Just like transcriptomic studies examining gene expression levels at the mRNA level, studies comparing the proteome of males and females also found remarkable sex differences (Arnold and Lusis, 2012). A recent human study revealed striking sex differences in serum metabolite concentrations, with 102 out of 131 metabolites differing significantly between males and females. This variation also correlated with specific genetic variants in metabolism-related genes (Mittelstrass et al., 2011). Thus, sexual dimorphism most commonly examined at the gene expression levels appear to be present also at the metabolomic and proteomic level.

### Epigenetic mechanism

There are known differences in epigenetic profiles of autosomal and sex chromosome genes between the sexes and in age groups (El-Maarri et al., 2007; Tobi et al., 2009; reviewed in Yuasa, 2010). Environmental and lifestyle factors also influence the epigenome (Brait et al., 2009; Bollati and Baccarelli, 2010; Waterland et al., 2010; Yuasa, 2010), some with sex-specificity (Tobi et al., 2009). Thus, epigenetic phenomena are likely to underlie the gender differences in cancer. There is currently no report implicating epigenetic changes in the sex disparity in cancer susceptibility, but future epigenetic epidemiology studies are likely to test these possibilities (Foley et al., 2009; Relton and Davey Smith, 2010). Epigenetic studies have also examined the tumor samples directly. An interesting finding was the male-specific hypermethylation of RASSF1A, but not the other genes under study (Vaissiere et al., 2009). Further examination of such clues is also likely to provide further insight into the gender differences in cancer susceptibility.

### Immune surveillance differences between males and females

Immune surveillance has now been recognized as a major physiologic mechanism protecting our bodies from cancer progression (Swann and Smyth, 2007; Eyles et al., 2010; Cramer and Finn, 2011). Since sexual dimorphism in immune response capacity is also well recognized, the differences in immune surveillance competence between males and females should contribute to the gender effect in cancer incidence. Generally, females mount higher innate and adaptive immune responses than males, which can result in faster clearance of pathogens but also contributes to increased susceptibility to inflammatory and autoimmune diseases in females compared with males. The expression of X-linked genes and microRNA (miRNA), as well as sex steroid hormones signaling through hormone receptors in immune cells can affect responses to immunological stimuli differently in males and females possibly resulting in male-female differential in incidence rates of not only cancer, but also infectious diseases, inflammatory disorders, autoimmune diseases, and vaccine responses (Klein, 2012).

#### Hormonal effects on immune surveillance

Both estrogens and androgens have been recognized as modulators of immune response and determinants of gender differences in disease susceptibility (Table 4; Olsen and Kovacs, 1996; Klein, 2012; Pennell et al., 2012). Sex hormones have been shown to modulate gene expression *in vitro* (cell line studies), and *in vivo* (gene expression profiling in animal and human cells and tissues). Their effects on target tissues are mediated by intracellular sex hormone receptors (Scheller et al., 1998; Kato et al., 2005; Heldring et al., 2007). Estrogen receptors (ERs) are present in B and T lymphocytes, neutrophils, macrophages, NK cells, bone marrow, and thymic stromal cells. Two types of ERs, ER-alpha and ER-beta have different functions despite having similar structures. Membrane-bound forms of ERs have different functions and are present on mature lymphocytes. ER-alpha is also expressed in dendritic cells, which can regulate the quality of the adaptive immune responses (Kovats, 2012). The effect of sex steroids on immune cell functions are generally achieved by their influences on cell signaling pathways, including nuclear factor-κB (NF-κB) and interferon regulatory factors (Kalaitzidis and Gilmore, 2005). Although not frequently mentioned, estrogen effects on the immune system may be dosage-dependent (Pennell et al., 2012).

**Table 4:** Effects of sex hormones on immune system*.

Estrogens	Androgens
*Overall*: enhanced immune response	*Overall*: diminished immune response
Enhanced antibody-mediated responses to exogenous antigens	Decreased antibody production
Enhanced T-cell cytotoxicity	Decreased T-cell proliferation
Increased cytokine and chemokine levels (including increased interferon-gamma production)	Alteration of cytokine profile of activated T-cells to decrease inflammation (including decreased interferon-gamma production)
Decreased IL-6 production	Increased IL-6 and IL-10 production
Enhanced antigen presenting cell activation	Decreased MHC class II antigen expression on antigen presenting cells
Stronger innate immune responses by promoting the differentiation of IFN-gamma-producing killer dendritic cells or by up-regulation of inducible nitric oxide synthase expression and nitric oxide production	Reduced inducible nitric oxide synthase expression and nitric oxide production

*Reviewed in Olsen and Kovacs (1996), Martin (2000), Lin et al. (2010), Ghazeeri et al. (2011), Karpuzoglu and Zouali (2011), Klein (2012), Pennell et al. (2012).

Besides regulation of immune cell activity through direct action mediated by their receptors, estrogen also selectively regulates regulatory miRNA expression in immune cells with functional consequences (Dai et al., 2008). Intracellular and membrane-bound androgen receptors are expressed in B and T lymphocytes (Sader et al., 2005). Non-gonadal (adrenal) steroids act through their glucocorticoid receptors which are strongly expressed by immune cells. It has been suggested that their sexually dimorphic actions may be linked to diseases with different prevalence in males and females (Duma et al., 2010). Besides, they show tissue-specific differences in their expression on immune cells. In addition to sex hormones’ contribution to sex differences, indirect effects can be mediated by growth hormone as well. For example, the well established sexual dimorphism in liver gene expression is due to growth hormone action (Clodfelter et al., 2006; Waxman and Holloway, 2009).

#### Sex chromosome-related effects on immune surveillance

Males have one copy of the X chromosome as opposed to females having two copies. To achieve gene dosage adjustment, X chromosome inactivation takes place. In each female cell, one of the X chromosomes is randomly inactivated. As a result, the same X chromosome is expressed in approximately 50% of female cells. If an X chromosome gene has a mutation or a deleterious polymorphism, all male cells will lack its protein product, but 50% of female cells may still have the functional protein provided that the other copy of the gene is functional. This is how an X chromosome gene mutations may have different effects on males and females and why this is usually the first explanation considered for a male-female differential in any physiologic phenomenon or a trait. Having just one copy of the X chromosome in males has been likened to the inbreeding depression where the protective effects of heterozygote advantage are lost (Morris and Harrison, 2009).

Identifying X chromosome-linked susceptibility causing gender disparity in cancer occurrence may be possible by examining large cancer registries in countries where excellent records exist and are linked to birth registries. In examination of cancer incidence in children of cancer cases, higher frequency of cancer in daughters of male cases compared with sons would implicate the contribution of an X chromosome gene to susceptibility. In an extensive survey of Denmark Cancer Registry, X chromosome-linked genetic factors were not found responsible for the excess risk of cancer in males (Biggar et al., 2009). However, these efforts aim to identify genetic risk factors for familial cancers, and negative findings do not rule out the presence of low penetrance genetic contributions to sporadic cancer occurrence.

The X chromosome contains the largest number of immune-related genes of the whole human genome (Fish, 2008; Libert et al., 2010; Pinheiro et al., 2011; Bianchi et al., 2012). Given the expression differences between males and females of the genes in sex chromosomes, it is expected that X chromosome-linked immune regulatory genes have important roles in mediating the sexual dimorphism in immune response. It has been shown that one of those genes, TLR7, is expressed at a higher level in females than in males with functional consequences (Berghofer et al., 2006). The X chromosome is not only enriched in immune regulatory genes, but also miRNAs with roles in the immune system (Pinheiro et al., 2011). The cumulative effects of the immune regulatory genes and miRNAs should be an important contributor to the differences in immune surveillance capacity between males and females underlying the differences in cancer susceptibility.

Growing evidence supports a regulatory cascade concept of sexual dimorphism in gene expression. This concept proposes that the initiating events of sexual differentiation during embryonic development trigger differential expression in many mediator genes that regulate the sexually dimorphic expression of downstream genes such as transcription factors and signaling molecules (Clodfelter et al., 2006; Bermejo-Alvarez et al., 2011). Epigenetic alteration mediated by sex chromosome genes is another mechanism for gender difference in autosomal gene expression (Wijchers and Festenstein, 2011). Sex chromosome genes can more directly contribute to sex differences too (Arnold and Burgoyne, 2004). Indirect evidence suggests a possible role of sex chromosome-linked genes in the regulation of sexual dimorphism. There is a generalized sex chromosome enrichment of the sexually dimorphic genes in all tissues examined, and a significant portion of common dimorphic genes across tissues are X- or Y-linked (Yang et al., 2006). Sex chromosome genes showing sex-biased expression may thus serve as candidate regulators (Yang et al., 2006).

The recently developed mouse model system used to identify effects of the sex chromosome complement without the confounding effects of differences in gonadal type has provided convincing evidence that sex chromosomes have effects on sexual dimorphism in autoimmunity independent of sex hormones (Palaszynski et al., 2005). This model system allows comparisons between XX and XY-(Sry-deficient) within a female hormonal background, as well as between XXSry and XY-Sry within a male hormonal background. The disease-promoting effect of the XX sex chromosome complement has been shown in this model for animal equivalents of multiple sclerosis and systemic lupus erythematosus. The expected sexual dimorphism in the immune system was also evident to a degree in this model which examines the correlations of genotypes with two different sex chromosome complements which exist in equivalent hormonal status (Smith-Bouvier et al., 2008). Thus, the female sex chromosome complement, as compared with the male sex chromosome complement, has a direct effect on promoting susceptibility to two autoimmune diseases with strong female predominance, and this effect is unlikely to be mediated by indirect effects of differences in sex hormones in this model. However, the earlier study showed that the XY-genotype is relatively immunostimulatory, compared with the XX genotype, confirming that it is the male hormone phenotype which is immunoinhibitory (Palaszynski et al., 2005).

In another cell line model, untreated male and female cells of the same age respond differently to the stressors, even before the production of fetal sex hormones (Penaloza et al., 2009). These findings also suggest independent effects of sex chromosomes, which is probably due to programming during early developmental stages (Bermejo-Alvarez et al., 2011).

### Genomic surveillance differences between males and females

As listed in Table 3, there are known physiologic differences between males and females that are relevant to the efficiency of genomic surveillance mechanisms. However, there is no clear pattern regarding why males have overall greater cancer susceptibility, or why females have higher incidence rates for few cancers. A recent review has noted that “too few studies are available to draw firm conclusions” (Kirsch-Volders et al., 2010).

### Molecular clues for gender differences in two cancers

Childhood ALL is more common in males, and examples of gender-specific associations have been presented (Table 2). One of the gender-specific associations concerns the interferon regulatory factor-4 (IRF4) locus (Do et al., 2010). An intronic SNP (rs12203592) shows a male-specific association (OR = 4.4, 95% CI = 1.5–12.6). In the experiments aimed to functionally replicate this association, the wildtype allele of the SNP showed a repressive effect on the transcription of IRF4. The variant allele, which is the risk allele for leukemia, lacked the repressive effect. This meant that the risk allele was associated with increased transcription of IRF4. IRF4 has a deleterious effect on leukemia and this result was biologically plausible. The explanation of the gender effect involved the influence of estrogen on the transcription factor NF-kappa B (NFkB), which regulates IRF4 transcription. Because estrogen has an inhibitory effect on NFkB, and males lack this effect, the combination of this with the possession of the variant allele were thought to be the reason for increased IRF4 production that results in the male-specific risk association. During childhood estrogen and androgen levels are very low and not much different between males and females (Ober et al., 2008). This suggests prenatal programming of sex hormone effect on autosomal gene expression. Likewise, the association of a MDM2 promoter region SNP with age-at-onset of childhood leukemia only in females (Do et al., 2009) suggests prenatal programming of gene expression regulation by estrogen signaling since this MDM2 association is attributed to the effect of this SNP on estrogen action on the gene expression (Bond et al., 2006).

Thyroid cancer is one of the few cancers that are more common in females. Not just the incidence, but also the tumor aggressiveness and prognosis show gender difference (Rahbari et al., 2010). While the exact mechanism of the female preponderance is unknown, androgen receptor expression in thyroid follicular cells through which androgens reduce proliferation of follicular cells may be a possible mechanism. Lacking this effect, females may be at higher risk of developing thyroid follicular cancer (Stanley et al., 2012). Furthermore, estrogens induce thyroid cancer cell proliferation and enhance anti-apoptotic potential (Lee et al., 2005). Despite possible involvement of sex hormones in thyroid cancer development, however, sex hormone gene variants failed to show any association with thyroid cancer risk (Schonfeld et al., 2012).

## Conclusion

The differences between males and females begin even before implantation of the zygote in the uterus. The differences continue throughout prenatal development phases, in childhood and adulthood. These differences generate a lot of contrasts, and variation in disease susceptibility is one of them. There are many common diseases that are more common in males or females. In cancer, susceptibility is generally higher in males although some cancers are more common in women. The same is true for autoimmunity in which females have an overall higher susceptibility, but males are more susceptible for few of them. The endogenous causes of the gender differences observed in diseases are largely unknown, and the situation in cancer research is not much different. Over the last decade, a lot of progress has been made in the field some of which are due to the advent of omics techniques. If the research community is convinced about the presence of gender differences in disease susceptibility, regardless of the techniques used, even more progress should be expected during the next decade. In this review, we presented data generated in different fields including descriptive and genetic epidemiology, physiology, and genetical genomics. Multidisciplinary efforts using the appropriate analytical approaches should unravel many remaining mysteries of gender differences in cancer susceptibility.

## Conflict of Interest Statement

The authors declare that the research was conducted in the absence of any commercial or financial relationships that could be construed as a potential conflict of interest.
